# Structure–efficiency relationship of access group antibiotics via SK chromatic descriptors

**DOI:** 10.3389/fchem.2026.1763932

**Published:** 2026-02-17

**Authors:** R. Rajambigai, T. Praveen, J. Ravi Sankar

**Affiliations:** Department of Mathematics, School of Advanced Sciences, Vellore Institute of Technology, Vellore, Tamil Nadu, India

**Keywords:** access group antibiotics, proper coloring, quantitative structure–property relationship analysis, regression models, SK chromatic indices

## Abstract

In graph theory, topological indices play a significant role as numerical descriptors of a graph, helping to summarize the physicochemical properties of a molecular graph. By capturing the molecular structure, they encode various aspects, including connectivity, complexity, molecular branching, and shape. Therefore, these indices are crucial in the initial stages of drug development for identifying potential drugs. In this study, quantitative structure–property relationship (QSPR) models were designed using SK chromatic indices to predict the physicochemical attributes of some access group antibiotics. Linear regression is used to analyze the physicochemical properties and the topological indices.

## Introduction

The AWaRe classification system was introduced by the World Health Organization (WHO) to guide the appropriate use of antibiotics and to prevent the development of antimicrobial resistance in the human body (World Health Organization, 2025). This system was launched in 2017 and is revised every 2 years. Antibiotics are classified into three groups, namely, Access, Watch, and Reserve, based on the priority in which they should be used (Cook and Wright, 2022). Access group antibiotics are first-line treatments used for common infections. These antibiotics are highly accessible, widely available, affordable, and have a high success rate. Watch group antibiotics are used only for specific infections and require careful monitoring. Reserve group antibiotics are used only as a last resort to treat life-threatening infections. Among all these groups, access to antibiotics is considered an important aspect because of their safe and effective clinical use. To gain a deeper understanding of access group antibiotics, we must comprehend their molecular and structural properties.

The chemical graph is a branch of graph theory that studies the molecular structure of drugs by transforming them into a molecular graph (Chartrand and Zhang, 2009). The molecular or chemical graph of a drug is a graph in which the vertices represent the atoms, and the edges between them represent the bonds connecting the atoms in the specific drug (Gao et al., 2016). Hydrogen atoms are neglected in the construction of molecular graphs, and the chemical bonds, irrespective of the multiplicity, are represented as single edges, thus forming simple underlying hydrogen-depleted graphs that focus on connectivity-based structural features. With the help of this, different topological indices are calculated to determine diverse features of molecular topology with an encrypted numerical parameter (Hayat and Asmat, 2023). Thus, topological indices are commonly used in the QSPR and QSAR analysis of different drugs (Lučić and Trinajstić, 1997). Wiener introduced the first topological index, the Wiener index, in 1947 (Wiener, 1947). It was first used to determine the physical characteristics of paraffin. Thereafter, other dimensions of topological indices were discovered, including the Randic index, the hyper-Wiener index, the connectivity index, and the Zagreb index, which have numerous applications in various fields (Gutman, 2013).

In recent years, topological indices have played a significant role in QSPR and QSAR investigations (Arockiaraj et al., 2024; Balasubramaniyan et al., 2024; Kour and Sankar, 2025). Much research has been done to find the use of chemical graph theory in QSPR analysis. Hakeem et al. (2024) studied the QSPR relationships among heart attack drugs through simple linear regression models using degree-based topological indices (Abdul et al., 2024). Parveen et al. (2022) analyzed some diabetes treatment drugs using a regression model and degree-based indices. Hosamani et al. (2017) used certain degree-based topological indices for QSPR analysis. Adnan et al. (2022) employed some degree-based topological indices to analyze some anti-tuberculosis drugs. Zhang et al. (2023) focused on computing regression models of some anti-malarial drugs using degree-based topological indices. Simran Kour *et al.* discussed a selection of tricyclic antidepressant drugs and anti-cancer drugs by a range of distance-based topological indices to understand their characteristics by integrating machine learning regression techniques (Kour and J., 2024; Kour and Sankar, 2025). Pandeeswari and Ravi Sankar (2025) compared two regression techniques to find the accuracy of their prediction by insighting topological indices into breast cancer drugs. Clement Johnson et al. used a zero divisor graph to find graph energy from its adjacency matrix and the Wiener index associated with the commutative rings from the zero divisor graph (Johnson and Sankar, 2023; Rayer and Jeyaraj, 2023). Sankar and Felix (2016) proposed a developed fuzzy decision-making trial and evaluation laboratory (DEMATEL) method and examined its effectiveness through some real-life applications. QSPR analysis using chromatic topological indices is a developing topic (Bommahalli Jayaraman and Balamurugan, 2025). Waqar Ali et al*.* introduced lower and extremal bounds for the second hyper-Zagreb and atom connectivity indices in trees with a fixed Roman domination number (Ali et al., 2025; Ali et al., 2024). Our research focuses on the molecular structures of access group antibiotics, which are hydrogen-depleted simple underlying molecular graphs. We are interested in the structural and functional properties of these compounds, and chromatic SK indices are used to analyze them (Shigehalli and Kanabur, 2016). The aim of the study is to evaluate the chromatic topological SK indices using proper colorings of 13 access group antibiotics, compute regression models to derive QSPR on the basis of the physicochemical properties, and validate the reliability of the models by comparing the measured values to the real ones. We obtain the topological descriptors of chemical graphs, characterize their molecular properties and correlations, and demonstrate the efficacy of this methodology to various classes of therapeutics.

## Materials and methods

**FIGURE 1:**
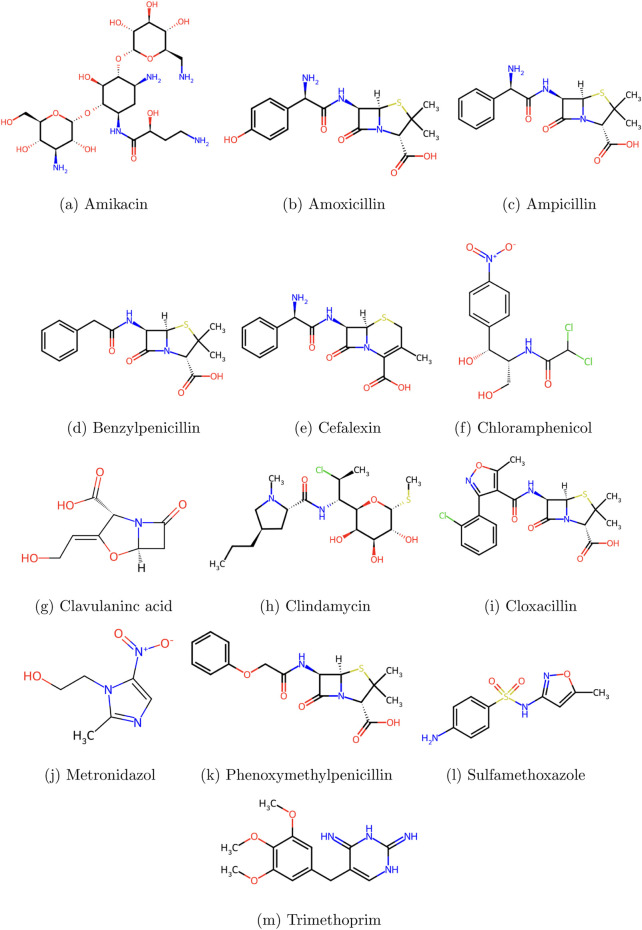
Access group antibiotics: **(a)** amikacin, **(b)** amoxicillin, **(c)** ampicillin, **(d)** benzylpenicillin, **(e)** cefalexin, **(f)** chloramphenicol, **(g)** clavulanic acid, **(h)** clindamycin, **(i)** cloxacillin, **(j)** metronidazole, **(k)** phenoxymethylpenicillin, **(l)** sulfamethoxazole, and **(m)** trimethoprim.

**FIGURE 2:**
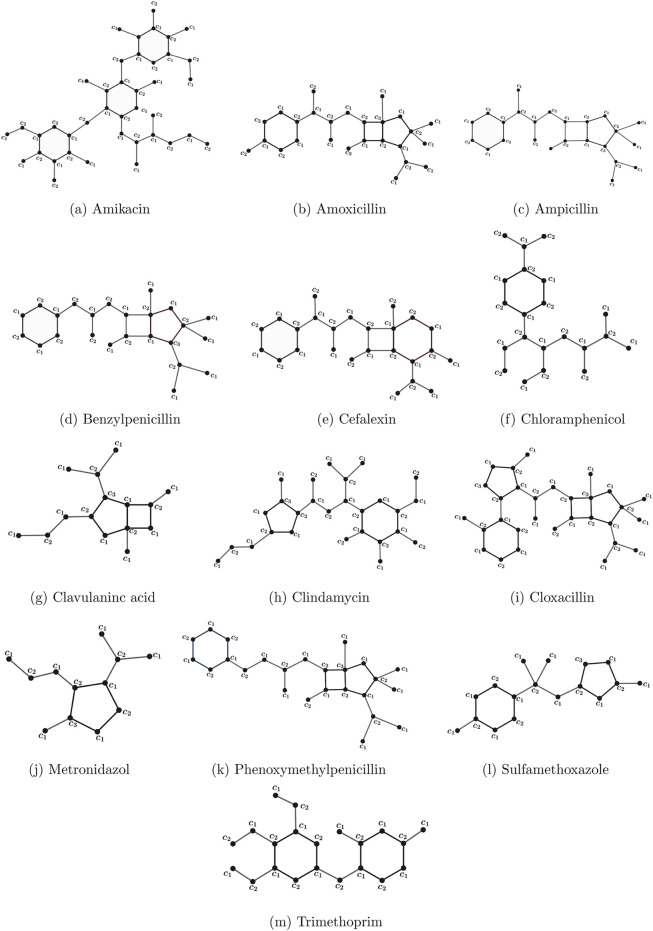
Proper coloring of molecular graphs of access group antibiotics: **(a)** amikacin, **(b)** amoxicillin, **(c)** ampicillin, **(d)** benzylpenicillin, **(e)** cefalexin, **(f)** chloramphenicol, **(g)** clavulanic acid, **(h)** clindamycin, **(i)** cloxacillin, **(j)** metronidazole, **(k)** phenoxymethylpenicillin, **(l)** sulfamethoxazole, and **(m)** trimethoprim.

Thirteen access group antibiotics were chosen for examination in this work, and their physicochemical characteristics were obtained from the PubChem (National Center for Biotechnology Information (NCBI), 2024) and ChemSpider databases (ChemSpider, 2025). Table 1 provides a comprehensive list of these medications together with their physicochemical characteristics, obtained from ChemSpider. Figure 1 displays the chemical structures of these medications, which were also obtained from ChemSpider. Figure 2 shows the proper coloring of the molecular graphs, which are constructed by treating the atoms in the molecule as vertices and the bonds between them as edges connecting their vertices using GeoGebra.

**TABLE 1:** Some access group antibiotics and their physical properties.

Drug	Density	Boiling point	Enthalpy	Flash point	Molar refractivity	Polarizabiliy	Surface tension	Molar volume
Amikacin	1.6	981.8	162.2	547.6	134.9	53.5	103.3	363.9
Amoxicillin	1.5	743.2	113.7	403.3	91.5	36.3	85.3	236.2
Ampicillin	1.5	683.9	105.4	367.4	89.9	35.7	74.3	239.3
Benzylpenicillin	1.4	663.3	102.5	355.0	86.3	34.2	67.9	235.2
Cefalexin	1.5	727.4	111.5	393.7	89.4	35.4	78.5	231.3
Chloramphenicol	1.5	644.9	100.0	343.8	72.6	28.8	66.1	208.8
Clavulanic acid	1.7	545.8	94.8	283.9	43.6	17.3	82.3	120.3
Clindamycin	1.3	628.1	106.5	333.6	107.9	42.8	56.2	327.2
Cloxacillin	1.6	689.7	106.2	370.9	106.2	42.1	79.2	279.3
Metronidazole	1.5	405.4	69.3	199.0	41.0	16.2	60.5	117.9
Phenoxymethylpenicillin	1.5	681.4	105.0	365.9	88.1	34.9	69.0	241.2
Sulfamethoxazole	1.5	482.1	74.7	245.4	62.5	24.8	70.9	173.1
Trimethoprim	1.3	405.2	65.7	198.8	75.5	29.9	45.7	220.8

### SK chromatic indices

The formula was introduced in our earlier unpublished work (Rajambigai, 2024)^1^.

$$SK^{\phi }\left(G\right)=\frac{1}{2}\underset{uv\in E\left(G\right)}{\sum }\left[c\left(u\right)+c\left(v\right)\right]=\frac{1}{2}\underset{1\leq t<s\leq \chi \left(G\right)}{\sum }\eta_{ts}\left[t+s\right],$$ (1)

$$SK_{1}^{\phi }\left(G\right)=\frac{1}{2}\underset{uv\in E\left(G\right)}{\sum }\left[c\left(u\right).c\left(v\right)\right]=\frac{1}{2}\underset{1\leq t<s\leq \chi \left(G\right)}{\sum }\left[ts\eta_{ts}\right],$$ (2)

$$SK_{2}^{\phi }\left(G\right)=\frac{1}{4}\underset{uv\in E\left(G\right)}{\sum }{\left[c\left(u\right)+c\left(v\right)\right]}^{2}=\frac{1}{4}\underset{1\leq t<s\leq \chi \left(G\right)}{\sum }\eta_{ts}{\left[t+s\right]}^{2},$$ (3)

In the Equations 1-3, t and s are colors in the set of colors $C=\left\{c_{1},c_{2},\ldots c_{l}\right\}$ and $\eta_{ts}$ denotes the edges having the colors t and s . Table 2 shows computed values of the SK chromatic indices of the 13 access group antibiotics by calculating the $\eta_{ts}$ values of the respective values and substituting in the above formula.

**TABLE 2:** SK chromatic indices of some access group antibiotics.

Chromatic topological index	$SK^{\phi }\left(G\right)$	$SK_{1}^{\phi }\left(G\right)$	$SK_{2}^{\phi }\left(G\right)$
Amikacin	63	42	94.5
Amoxicillin	45	33	74.5
Ampicillin	41.5	30.5	68.25
Benzylpenicillin	41.5	30.5	68.25
Cefalexin	40.5	27	60.75
Chloramphenicol	30	20	45
Clavulanic acid	26.5	20.5	45.75
Clindamycin	44	31	70.5
Cloxacillin	54	40.5	91.5
Metronidazole	20	15	34.5
Phenoxymethylpenicillin	43.5	32	72.25
Sulfamethoxazole	28.5	20.5	46.25
Trimethoprim	33	22	49.5

## Results

The eight physicochemical properties mentioned in Table 1 are used in this study. The resulting formula is used to determine correlations between relevant chromatic topological indices and various physicochemical characteristics of access group antibiotics. The linear regression model employed in this article is

$$P=a+b\left(TI^{\phi }\right).$$ (4)

In the above formula, P represents the physicochemical properties of the listed drugs, $TI^{\phi }$ is the calculated chromatic topological index values of the respective drugs, A is a constant term, and b is the regression coefficient. The values of A and b are computed via SPSS software and Microsoft Excel by evaluating the physicochemical properties and the chromatic topological index values across the 13 access group antibiotics. Here, the physicochemical properties of the drugs are considered as dependent values, and the chromatic topological indices of the molecular graphs of the access group antibiotics are considered as independent variables. By applying Equation 4, the linear regression model for the previously mentioned chromatic topological indices is expressed in Table 3.

**TABLE 3:** Regression models for SK chromatic index.

Density	$1.459+0.001\left(SK^{\phi }\right)$	$1.439+0.002\left(SK_{1}^{\phi }\right)$	$1.441+0.001\left(SK_{2}^{\phi }\right)$
Boiling point	$189.2+11.36\left(SK^{\phi }\right)$	$199.8+15.55\left(SK_{1}^{\phi }\right)$	$200.8+6.885\left(SK_{2}^{\phi }\right)$
Enthalpy	$32.48+1.747\left(SK^{\phi }\right)$	$34.77+2.368\left(SK_{1}^{\phi }\right)$	$34.88+1.049\left(SK_{2}^{\phi }\right)$
Flash point	$68.23+6.871\left(SK^{\phi }\right)$	$74.61+9.407\left(SK_{1}^{\phi }\right)$	$75.24+4.164\left(SK_{2}^{\phi }\right)$
Molar refractivity	$0.6598+2.109\left(SK^{\phi }\right)$	$3.192+2.867\left(SK_{1}^{\phi }\right)$	$2.890+1.277\left(SK_{2}^{\phi }\right)$
Polarizability	$0.2302+0.8369\left(SK^{\phi }\right)$	$1.231+1.138\left(SK_{1}^{\phi }\right)$	$1.112+0.5068\left(SK_{2}^{\phi }\right)$
Surface tension	$44.37+0.7072\left(SK^{\phi }\right)$	$43.83+1.011\left(SK_{1}^{\phi }\right)$	$44.05+04449\left(SK_{2}^{\phi }\right)$
Molar volume	$17.05+5.411\left(SK^{\phi }\right)$	$26.11+7.264\left(SK_{1}^{\phi }\right)$	$24.99+3.241\left(SK_{2}^{\phi }\right)$

### Relationship between correlation coefficients and physicochemical properties of the drugs

**FIGURE 3:**
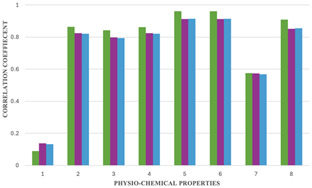
Graphical representation between correlation coefficients and physiochemical properties of the drugs.

In this study, the correlations of the chromatic SK indices and the eight physicochemical properties are listed in Table 4; the correlations that are strong are highlighted in bold, and the relationship is graphically represented in Figure 3. The correlation coefficients were generated to assess the link between molecular characteristics and chromatic topological indices. All statistical computations, including the calculation of correlation coefficients, were performed using Microsoft Excel (Microsoft Corporation).

**TABLE 4:** Correlation coefficients of physical properties.

Chromatic topological index	Density	Boiling point	Enthalpy	Flash point	Molar refractivity	Polarizability	Surface tension	Molar volume
$SK^{\phi }$	0.0882	**0.8619**	**0.8427**	**0.8619**	**0.9609**	**0.9609**	0.5746	**0.9080**
$SK_{1}^{\phi }$	0.1380	**0.8244**	**0.7979**	**0.8244**	**0.9125**	**0.9126**	0.5739	**0.8532**
$SK_{2}^{\phi }$	0.1322	**0.8205**	**0.7948**	**0.8205**	**0.9138**	**0.9139**	0.5680	**0.8559**

Significant values are highlighted in bold.

### Assessment of statistical metrics and standard estimation error

The statistical metrics are integrated for all chromatic topological indices and physicochemical properties in Tables 5–7 to help us understand the relationship among them. The statistical parameters for all the chromatic topological indices, including the sample size N , the constant term A , the slope b , the percentage of dependent variables $R^{2}$ , Fisher’s statistic, the significance value p , and the significance of the relationship, are observed. In terms of interpretation, a p -value less than 0.05 is deemed statistically significant; a p -value greater than 0.05 is considered an absence of statistical significance. These metrics not only allow comparisons but also give well-informed calculations. Table 8 lists the standard estimation error for the physicochemical properties of the access group antibiotics. The calculation of standard estimation error improves the accuracy of predictions from QSPR models. Tables 9–16 show the comparison between the original and calculated values of physicochemical properties from regression models of the SK chromatic indices.

**TABLE 5:** Statistical metrics integrated into the QSPR modeling framework for the $SK^{\phi }$ index.

Property	N	A	b	$R^{2}$	*p*	F	Indicator
Density	13	1.459	0.001	0.0078	0.7731	0.087	Non-significant
Boiling point	13	189.2	11.36	0.7447	0.0001	32.08	Significant
Enthalpy	13	32.48	1.747	0.7095	0.0003	26.86	Significant
Flash point	13	68.23	6.871	0.7445	0.0001	32.06	Significant
Molar refractivity	13	0.659	2.109	0.9234	0.0000	132.5	Significant
Polarizability	13	0.230	0.836	0.9234	0.0000	132.6	Significant
Surface tension	13	44.37	0.707	0.3303	0.0399	5.425	Significant
Molar volume	13	17.05	5.411	0.8257	0.0000	52.17	Significant

**TABLE 6:** Statistical metrics integrated into the QSPR modeling framework for the $SK_{1}^{\phi }$ index.

Property	N	A	b	$R^{2}$	*p*	F	Indicator
Density	13	1.439	0.002	0.0191	0.6528	0.214	Non-significant
Boiling point	13	199.8	15.55	0.6798	0.0005	23.36	Significant
Enthalpy	13	34.77	2.368	0.6349	0.0011	19.13	Significant
Flash point	13	74.61	9.407	0.6798	0.0005	23.35	Significant
Molar refractivity	13	3.192	2.867	0.8311	0.0000	54.15	Significant
Polarizability	13	1.231	1.138	0.8314	0.0000	54.25	Significant
Surface tension	13	43.83	1.011	0.3286	0.0405	5.384	Significant
Molar volume	13	26.11	7.264	0.7249	0.0002	28.98	Significant

**TABLE 7:** Statistical metrics integrated into the QSPR modeling framework for the $SK_{2}^{\phi }$ index.

Property	N	A	b	$R^{2}$	*p*	F	Indicator
Density	13	1.441	0.001	0.0176	0.6662	0.197	Non-significant
Boiling point	13	200.8	6.885	0.6738	0.0006	22.72	Significant
Enthalpy	13	34.88	1.049	0.6303	0.0012	18.75	Significant
Flash point	13	75.24	4.164	0.6737	0.0006	22.72	Significant
Molar refractivity	13	2.890	1.277	0.8339	0.0000	55.23	Significant
Polarizability	13	1.112	0.507	0.8342	0.0000	55.33	Significant
Surface tension	13	44.05	0.445	0.3221	0.0431	5.227	Significant
Molar volume	13	24.99	3.241	0.7299	0.0002	29.72	Significant

**TABLE 8:** Standard error estimation for some physical properties of the drugs.

Drug	Density	Boiling point	Enthalpy	Flash point	Molar refractivity	Polarizability	Surface tension	Molar volume
$SK^{\phi }$	0.1160	81.7306	13.6715	49.4409	7.4429	2.9558	12.3310	30.2753
$SK_{1}^{\phi }$	0.1154	91.2377	15.3054	55.1895	10.9908	4.3402	12.3372	38.0288
$SK_{2}^{\phi }$	0.1155	92.1425	15.4069	55.7371	10.9120	4.3275	12.3989	37.6972

**TABLE 9:** Comparison between the original and calculated density values from regression models of chromatic topological indices.

Drug	Density (in $g/cm^{3}$ )	$SK^{\phi }\left(G\right)$	$SK_{1}^{\phi }\left(G\right)$	$SK_{2}^{\phi }\left(G\right)$
Amikacin	1.6±0.1	1.51	1.52	1.52
Amoxicillin	1.5±0.1	1.49	1.50	1.50
Ampicillin	1.5±0.1	1.49	1.49	1.49
Benzylpenicillin	1.4±0.1	1.49	1.49	1.49
Cefalexin	1.5±0.1	1.49	1.49	1.49
Chloramphenicol	1.5±0.1	1.48	1.48	1.48
Clavulanic acid	1.7±0.1	1.48	1.48	1.48
Clindamycin	1.3±0.1	1.49	1.49	1.49
Cloxacillin	1.6±0.1	1.50	1.52	1.51
Metronidazole	1.5±0.1	1.48	1.47	1.47
Phenoxymethylpenicillin	1.5±0.1	1.49	1.49	1.49
Sulfamethoxazole	1.5±0.1	1.48	1.48	1.48
Trimethoprim	1.3±0.1	1.49	1.48	1.48

**TABLE 10:** Comparison between the original and calculated boiling point values from regression models of chromatic topological indices.

Drug	Boiling point (at 760 mmHg)	$SK^{\phi }\left(G\right)$	$SK_{1}^{\phi }\left(G\right)$	$SK_{2}^{\phi }\left(G\right)$
Amikacin	981.8±65.0	904.9	852.9	851.4
Amoxicillin	743.2±60.0	700.4	712.9	713.7
Ampicillin	683.9±55.0	660.6	674.0	670.7
Benzylpenicillin	663.3±55.0	660.6	674.1	670.7
Cefalexin	727.4±60.0	649.3	619.7	619.1
Chloramphenicol	644.9±55.0	530.0	510.8	510.6
Clavulanic acid	545.8±50.0	490.2	518.6	515.8
Clindamycin	628.1±55.0	689.0	681.9	686.2
Cloxacillin	689.7±55.0	802.6	829.6	830.8
Metronidazole	405.4±25.0	416.4	433.1	438.3
Phenoxymethylpenicillin	681.4±55.0	683.4	697.4	698.2
Sulfamethoxazole	482.1±55.0	512.9	518.6	519.2
Trimethoprim	405.2±55.0	564.1	541.9	541.6

**TABLE 11:** Comparison between the original and calculated enthalpy values from regression models of chromatic topological indices.

Drug	Enthalpy (KJ/mol)	$SK^{\phi }\left(G\right)$	$SK_{1}^{\phi }\left(G\right)$	$SK_{2}^{\phi }\left(G\right)$
Amikacin	162.2±6.0	142.54	134.22	134.01
Amoxicillin	113.7±3.0	111.09	112.91	113.03
Ampicillin	105.4±3.0	104.98	106.99	106.47
Benzylpenicillin	102.5±3.0	104.98	106.99	106.47
Cefalexin	111.5±3.0	103.23	98.71	98.61
Chloramphenicol	100.0±3.0	84.89	82.13	82.09
Clavulanic acid	94.8±6.0	78.78	83.31	82.87
Clindamycin	106.5±6.0	109.35	108.18	82.09
Cloxacillin	106.2±3.0	126.82	130.67	130.86
Metronidazole	69.3±3.0	67.42	70.29	71.07
Phenoxymethylpenicillin	105.0±3.0	108.47	110.55	110.67
Sulfamethoxazole	74.7±3.0	82.27	83.31	83.39
Trimethoprim	65.7±3.0	90.13	86.87	86.81

**TABLE 12:** Comparison between the original and calculated flash point values from regression models of chromatic topological indices.

Drug	Flash point (in °C )	$SK^{\phi }\left(G\right)$	$SK_{1}^{\phi }\left(G\right)$	$SK_{2}^{\phi }\left(G\right)$
Amikacin	547.6±34.3	501.10	469.70	468.74
Amoxicillin	403.3±32.9	377.43	385.04	385.46
Ampicillin	367.4±31.5	353.38	361.52	359.43
Benzylpenicillin	355.0±31.5	353.38	361.52	359.43
Cefalexin	393.7±32.9	346.51	328.59	328.20
Chloramphenicol	343.8±31.5	274.36	262.75	262.62
Clavulanic acid	283.9±30.1	250.31	267.45	265.74
Clindamycin	333.6±31.5	370.55	366.23	368.80
Cloxacillin	370.0±31.5	439.26	455.59	456.25
Metronidazole	199.0±23.2	205.65	215.72	218.89
Phenoxymethylpenicillin	365.9±31.5	367.12	375.65	376.09
Sulfamethoxazole	245.4±31.5	264.05	267.45	267.83
Trimethoprim	198.8±31.5	294.97	281.56	281.36

**TABLE 13:** Comparison between the original and calculated molar refractivity values from regression models of chromatic topological indices.

Drug	Molar refractivity (in $cm^{3}$ )	$SK^{\phi }\left(G\right)$	$SK_{1}^{\phi }\left(G\right)$	$SK_{2}^{\phi }\left(G\right)$
Amikacin	134.9±0.4	133.53	123.61	123.57
Amoxicillin	91.5±0.4	95.56	97.80	98.03
Ampicillin	89.9±0.4	88.18	90.64	90.05
Benzylpenicillin	86.3±0.4	88.18	90.64	90.05
Cefalexin	89.4±0.4	86.07	80.60	80.47
Chloramphenicol	72.6±0.3	63.93	60.53	60.36
Clavulanic acid	43.6±0.4	56.55	61.97	61.31
Clindamycin	107.9±0.4	93.46	92.07	92.92
Cloxacillin	106.2±0.4	114.55	119.31	119.74
Metronidazole	41.0±0.5	42.84	46.19	46.95
Phenoxymethylpenicillin	88.1±0.4	92.40	94.94	95.15
Sulfamethoxazole	62.5±0.4	60.77	61.97	61.95
Trimethoprim	75.5±0.5	70.26	66.27	66.10

**TABLE 14:** Comparison between the original and calculated polarizability values from regression models of chromatic topological indices.

Drug	Polarizability (in $10^{-24}\;cm^{3}$ )	$SK^{\phi }\left(G\right)$	$SK_{1}^{\phi }\left(G\right)$	$SK_{2}^{\phi }\left(G\right)$
Amikacin	53.5±0.5	52.96	49.03	49.01
Amoxicillin	36.3±0.5	37.89	38.79	38.87
Ampicillin	35.7±0.5	34.96	35.94	35.70
Benzylpenicillin	34.2±0.5	34.96	35.94	35.70
Cefalexin	35.4±0.5	34.12	31.96	31.90
Chloramphenicol	28.8±0.5	25.34	23.99	23.92
Clavulanic acid	17.3±0.5	22.41	24.56	24.29
Clindamycin	42.8±0.5	37.05	36.51	36.84
Cloxacillin	42.1±0.5	45.42	47.32	47.48
Metronidazole	16.2±0.5	16.97	18.30	18.59
Phenoxymethylpenicillin	34.9±0.5	36.64	37.65	37.73
Sulfamethoxazole	24.8±0.5	24.08	24.56	24.55
Trimethoprim	29.9±0.5	27.85	26.27	26.19

**TABLE 15:** Comparison between the original and calculated surface tension values from regression models of chromatic topological indices.

Drug	Surface tension (in dyne/cm )	$SK^{\phi }\left(G\right)$	$SK_{1}^{\phi }\left(G\right)$	$SK_{2}^{\phi }\left(G\right)$
Amikacin	103.3±5.0	88.92	86.29	86.09
Amoxicillin	85.3±5.0	76.19	77.19	77.19
Ampicillin	74.3±5.0	73.72	74.67	74.41
Benzylpenicillin	67.9±5.0	73.72	74.67	74.41
Cefalexin	78.5±5.0	73.01	71.13	71.08
Chloramphenicol	66.1±3.0	65.59	64.05	64.07
Clavulanic acid	82.3±5.0	63.11	64.56	64.40
Clindamycin	56.2±5.0	75.49	75.17	75.42
Cloxacillin	79.2±5.0	82.56	84.78	84.76
Metronidazole	60.5±7.0	58.51	58.99	59.39
Phenoxymethylpenicillin	69.0±5.0	75.13	76.18	76.19
Sulfamethoxazole	70.9±3.0	64.53	64.56	64.63
Trimethoprim	45.7±7.0	67.71	66.07	66.07

**TABLE 16:** Comparison between the original and calculated molar volume values from regression models of chromatic topological indices.

Drug	Molar volume (in $cm^{3}$ )	$SK^{\phi }\left(G\right)$	$SK_{1}^{\phi }\left(G\right)$	$SK_{2}^{\phi }\left(G\right)$
Amikacin	363.9±5.0	357.94	331.19	331.27
Amoxicillin	236.2±5.0	260.55	265.82	266.44
Ampicillin	239.3±3.0	241.61	247.66	246.19
Benzylpenicillin	235.2±5.0	241.61	247.66	246.19
Cefalexin	231.3±5.0	236.19	222.24	221.88
Chloramphenicol	208.8±3.0	179.38	171.39	170.84
Clavulanic acid	120.3±5.0	160.44	175.02	173.27
Clindamycin	327.2±5.0	255.13	251.29	253.48
Cloxacillin	279.3±5.0	309.24	320.30	321.54
Metronidazole	117.9±7.0	125.27	135.07	136.80
Phenoxymethylpenicillin	241.2±5.0	252.43	258.56	259.15
Sulfamethoxazole	173.1±3.0	171.26	175.02	174.89
Trimethoprim	220.8±7.0	195.61	185.92	185.42

## Discussion

In this study, we investigated the correlation between the three chromatic topological indices and eight physicochemical properties of 13 access group antibiotics. The data listed in Table 3 show the correlation coefficients, especially the strong correlations ( r>0.7 are highlighted in bold) noted between three chromatic topological indices and six physicochemical attributes. Upon examining all, the $SK^{\phi }$ index illustrated the strongest correlation with the following properties: boiling point (r=0.8619) , enthalpy (r=0.8427) , flash point (r=0.8619) , molar refractivity (r=0.9609) , polarizability (r=0.9690) , and molar volume (r=0.9080) , which shows that this chromatic topological index may be an effective predictor of these molecular properties. On the other hand, all the chromatic topological indices show weak correlation (r<0.2) with density, thus having a trivial impact on this physicochemical property.

The QSPR analysis using the statistical attributes such as $R^{2}$ , F -statistic, p -value, and regression coefficient b are listed in Tables 5–7. Both molar refractivity and polarizability show strong dependency on the index $SK^{\phi }$ . Notably, molar refractivity has $R^{2}=0.9234$ , p=0.00 , and F=132.5 with the $SK^{\phi }$ index, and polarizability has $R^{2}=0.9234$ , p=0.00 , and F=132.6 with the $SK^{\phi }$ index. Similarly, the molar volume, boiling point, flash point, and enthalpy show moderate dependency on $SK^{\phi }$ . Molar volume has $R^{2}=0.8257$ , p=0.00 , and F=52.17 with the $SK^{\phi }$ index. Boiling point has $R^{2}=0.7447$ , p=0.0001 , and F=32.01 with the $SK^{\phi }$ index. Flash point has $R^{2}=0.7445$ , p=0.0001 , and F=32.06 with the $SK^{\phi }$ index, and enthalpy has $R^{2}=0.7095$ , p=0.0003 , and F=26.86 with the $SK^{\phi }$ index. The density and surface tension show weak dependency. This analysis shows that the values of the chromatic SK index are necessary to understand the property of interest, such as polarizability, which helps elucidate the intermolecular forces and supports designing models with specific dielectric properties, thus playing a crucial role in model development.

Similarly, the values of the chromatic SK index for predicting molar refractivity and enthalpy can also be used for drug design, although the estimated error is slightly high. Because density and surface tension have a weak correlation, more advanced techniques may be needed, such as non-linear models or hybrid modeling, for more accuracy. Therefore, the study demonstrates the need to modify QSPR models to define physicochemical attributes to improve predictions and guide future work.

## Conclusion

Three coloring-based topological indices have been used to characterize the structural attributes of some access group antibiotics. QSPR analysis has also been done on the spatial arrangement of atoms and the physicochemical characteristics of certain drugs. It is found that some chromatic topological indices are effective at predicting properties, such as polarizability, molar refractivity, and enthalpy. Therefore, the study shows that molecular structure plays an important role in determining the properties of certain drugs, indicating that chromatic topological indices are necessary for predicting such properties. This technique helps speed drug development by enabling efficient identification of suitable drugs, thereby minimizing the need for research facilities.

## Data Availability

The original contributions presented in the study are included in the article/Supplementary Material; further inquiries can be directed to the corresponding author.

## Footnotes

1
*Chromatic SK indices of certain flower graphs*. Manuscript submitted for publication.
